# Genes and genetic mechanisms contributing to fall armyworm resistance in maize

**DOI:** 10.1002/tpg2.20311

**Published:** 2023-03-02

**Authors:** Marilyn L. Warburton, Sandra W. Woolfolk, J. Spencer Smith, Leigh K. Hawkins, Lina Castano‐Duque, Matthew D. Lebar, W. Paul Williams

**Affiliations:** ^1^ USDA ARS Plant Germplasm Introduction and Testing Research Unit Pullman WA USA; ^2^ USDA ARS Corn Host Plant Resistance Research Unit Mississippi State MS USA; ^3^ USDA ARS Food and Feed Safety Research Unit New Orleans LA USA

## Abstract

Maize (*Zea mays* L.) is a crop of major economic and food security importance globally. The fall armyworm (FAW), *Spodoptera frugiperda*, can devastate entire maize crops, especially in countries or markets that do not allow the use of transgenic crops. Host‐plant insect resistance is an economical and environmentally benign way to control FAW, and this study sought to identify maize lines, genes, and pathways that contribute to resistance to FAW. Of the 289 maize lines phenotyped for FAW damage in artificially infested, replicated field trials over 3 years, 31 were identified with good levels of resistance that could donate FAW resistance into elite but susceptible hybrid parents. The 289 lines were genotyped by sequencing to provide single nucleotide polymorphism (SNP) markers for a genome‐wide association study (GWAS), followed by a metabolic pathway analysis using the Pathway Association Study Tool (PAST). GWAS identified 15 SNPs linked to 7 genes, and PAST identified multiple pathways, associated with FAW damage. Top pathways, and thus useful resistance mechanisms for further study, include hormone signaling pathways and the biosynthesis of carotenoids (particularly zeaxanthin), chlorophyll compounds, cuticular wax, known antibiosis agents, and 1,4‐dihydroxy‐2‐naphthoate. Targeted metabolite analysis confirmed that maize genotypes with lower levels of FAW damage tend to have higher levels of chlorophyll a than genotypes with high FAW damage, which tend to have lower levels of pheophytin, lutein, chlorophyll b and β‐carotene. The list of resistant genotypes, and the results from the genetic, pathway, and metabolic study, can all contribute to efficient creation of FAW resistant cultivars.

## Core Ideas

- Genome‐wide association study and metabolic pathway analysis identified genes and pathways associated with fall armyworm resistance.
- 31 maize lines with good resistance to fall armyworm were identified that can be useful donor lines.
- Pathways identified may provide evidence of new resistance mechanisms for direct selection or new research avenues.
- Measurement of targeted metabolites confirms pathway analysis results and provide further avenues of new research.

**Abbreviations**
BLUE: best linear unbiased estimator
CHPRRU: Corn Host Plant Resistance Research Unit
CIMMYT: International Maize and Wheat Improvement Center, in Spanish
FAW: fall armyworm
GBS: genotyping by sequencing
GLM: general linear model
GWAS: genome‐wide association study
MAF: minor allele frequency
MLM: mixed linear model
PAST: pathway analysis study tool
QTL: quantitative trait loci

## 1 INTRODUCTION

Maize (*Zea mays* L.) provides food, feed, and industrial ingredients to the economy of the United States, worth many billions of dollars annually. It is a major staple food crop globally as well, and total grain production was 1266 million tons in 2019 (World Data Atlas, Knoema.com). The fall armyworm (FAW), *Spodoptera frugiperda* (J. E. Smith) (Lepidoptera: Noctuidae), originally evolved with maize in the tropical and subtropical regions of the Americas, and although it feeds on a broad range of host plants, it prefers gramineous plants including maize, and can devastate a maize crop in a short time (Overton et al., 2021). It has now spread to almost every country of the Americas, Africa, and Asia as an invasive and destructive species (Goergen et al., 2016). This is of particular concern in Africa, where many countries do not allow the use of transgenic crops, removing some insect control options from farmers, but new genetic editing techniques may be more acceptable to the public (Turnbull et al., 2021).

Native resistance, or host‐plant insect resistance conferred by naturally occurring maize genes, is an economical and environmentally benign way to control FAW. Maize lines containing native resistance genes were created in the 1970s through 1990s by the International Maize and Wheat Improvement Center (CIMMYT) in Mexico (Mihm et al., 1988). The USDA‐ARS Corn Host Plant Resistance Research Unit (CHPRRU) developed and released several resistant temperate maize inbred lines; these include Mp496, Mp701–708, Mp713, Mp714, and Mp716 (Scott & Davis, 1981; Scott et al., 1982; Williams & Davis, 1980, 1982, 2000, 1984, 2002; Williams et al., 1990). Although high levels of FAW infestation are better controlled by transgenic hybrids containing the Bt gene than conventional hybrids with native resistance, both transgenic and conventionally bred resistant maize hybrids are significantly less damaged by FAW feeding than non‐resistant commercial hybrids (Williams et al., 1997).

**Core Ideas**

- Genome‐wide association study and metabolic pathway analysis identified genes and pathways associated with fall armyworm resistance.
- 31 maize lines with good resistance to fall armyworm were identified that can be useful donor lines.
- Pathways identified may provide evidence of new resistance mechanisms for direct selection or new research avenues.
- Measurement of targeted metabolites confirms pathway analysis results and provide further avenues of new research.

Native resistance to insects in maize is generally quantitative in nature, governed by many genes, rather than a single gene conferring most or all of the resistance (McMullen et al., 2009). Quantitative trait loci (QTL) for FAW resistance in maize have been identified including one with a very large phenotypic effect in bin 9.03 (Brooks et al., 2007; Womack et al., 2018; 2020). Genetic mechanisms causing FAW resistance in maize can be due to morphological features such as structural barriers (waxes or tougher cell walls); antibiosis, or the creation of metabolites toxic or off‐putting to the insects; or hormones that call insect predators (McMullen et al., 2009). A previous study by Davis et al. (1998) found that resistance in young plants was linked to thickness of the cuticle and the epidermal cell wall complex, which was nearly twice as thick in resistant plants than in susceptible plants and contained more hemicellulose. In addition, the inner whorl tissue from the resistant inbreds were tougher than in the susceptible, and resistant hybrids completed the transition from juvenile to adult tissues (which are less palatable to insects) sooner than susceptible hybrids. FAW larvae feeding on maize plants in the whorl stage of growth feed primarily on the yellow‐green portion of whorl leaves. Davis et al. (1999) compared the sizes of larvae fed the yellow‐green leaves of resistant or susceptible hybrids, compared to the outer (green) or innermost (yellow‐white) portions of the leaves. Larvae feeding on the susceptible hybrid were larger than those feeding on the resistant, and larvae feeding on the yellow‐green leaves in resistant (and to a lesser extent, susceptible) plants were smaller compared to those feeding on other portions within the whorl. Thus, it is hypothesized that there are antibiosis compounds localized there, especially in resistant plants, that protect the growing point of the seedling plants (Williams et al., 1998).

Both QTL mapping and association mapping may identify genes and genomic regions associated with a trait of interest (Jamann et al., 2015; Xiao et al., 2017). A QTL mapping experiment will likely identify very long genomic regions associated with the trait and will only find the alleles that happen to be segregating in the two parents of the mapping population. Conversely, a genome‐wide association study (GWAS) will identify far more regions associated with a trait, often precisely enough to narrow down to one or a few genes influencing the trait, but rarely identifies large effect genes for highly quantitative traits, and many genes may be missed (Flint‐Garcia et al., 2005). The problems of gene identification associated with QTL and GWAS methods may be remedied using a metabolic pathway analysis of the output of the GWAS. A pathway analysis identifies the genes linked to SNPs that GWAS had found to be associated with the traits of interest. These genes are then assigned to the metabolic pathways they are known to be a part of, using published gene annotation information. The degree of association of the pathways to the traits is calculated, leading to a new understanding of how the trait develops in the plants. The pathway analysis may identify more useful genes than the GWAS, and these genes will be grouped into pathways of known function (Tang et al., 2015; Thrash et al., 2020a, 2020b).

The spread of FAW outside its native range has increased the importance of identifying genes and regions of the maize genome contributing resistance to FAW both to facilitate breeding of non‐GMO lines with FAW resistance, and to identify genes as candidates for gene editing. Thus, this study was undertaken to dissect the genetic basis and physiological mechanisms of resistance to FAW in maize. The objectives of this study were to identify resistant maize lines and SNPs associated with FAW resistance via GWAS; to use this information to identify metabolic pathways associated with FAW resistance via Pathway Association Study Tool (PAST); validate the presence of one or more of these metabolites in resistant versus susceptible plants; to integrate these pathways into specific mechanisms used by maize plants as protection from FAW; and to compare these results to previous genomic and metabolomic studies of insect resistance in maize.

## 2 MATERIALS AND METHODS

### 2.1 Germplasm and data generation

A panel of 289 diverse maize inbred lines was used for this study (Table S1). The panel included both FAW resistant and susceptible lines from various breeding programs, some of which formed association panels published previously (Flint‐Garcia et al., 2005; Warburton et al., 2013). These lines were genotyped and phenotyped as inbreds. They were grown at the R. R. Foil Plant Science Research Center, Mississippi State, Mississippi (MS) in 2019, 2020, and 2021. Seeds were planted in 4 m single‐row plots with 0.97 m spacing between rows and thinned to 20 plants per plot. The experiment was planted in a randomized complete block design with two replications in 2019 and 2020 and three replications in 2021. Standard production practices were followed, and supplemental irrigation was applied as needed.

The SAW neonate larvae used in this study were obtained from the laboratory colony at the insect rearing facility of the CHPRRU, USDA‐ARS, Mississippi State. The larvae were reared using artificial diet following the procedure described by Davis (1989). Within 24 h following the egg hatch, first instar larvae (neonates) were mixed with corn cob grits. The mixture was dispensed into the maize whorl utilizing a mechanical applicator (Wiseman et al., 1980). Each individual plant was infested with approximately 40 FAW neonates at the mid‐whorl stage (V‐7 of maize growth stage). All plants within experimental plots were rated individually to determine the leaf feeding damage caused by FAW. The damage was visually rated at 7 and 14 days after infestation. The 7‐day and 14‐day leaf feeding ratings were conducted on a 0 (no visible leaf feeding damage) to 9 scale according to Davis et al. (1992; Table S2). The 7‐day ratings were conducted by one person and the 14‐day ratings were conducted independently by two people.

The mean rating for each entry was calculated as an adjusted mean (best linear unbiased estimator; BLUE) using mixed model methodology in the PROC GLIMMIX procedure of SAS 9.4 (SAS Institute, Cary, NC) and can be found in Table S1. For the 7‐day ratings, the individual plants within a plot were treated as subsamples. Entry means were calculated within years treating genotype as a fixed effect and replication and genotype by replication as random effects. To combine the 7‐day ratings over years, genotype was treated as a fixed effect and year, replication nested within year, year by genotype interaction, and genotype by replication nested in year were all treated as random effects. For the 14‐day ratings two sets of individual plant ratings, one set per rater, were used as subsamples for each plot. Entry means were calculated similarly to the 7‐day ratings, but additional model terms were added to account for the variance due to different raters. Within years, genotype was treated as a fixed effect and replication, rater, rater by replication, genotype by replication, and genotype by rater by replication were all treated as random effects. When the data was combined across 3 years, genotype was treated as a fixed effect and year, replication nested in year, rater, rater by replication nested in year, year by genotype interaction, year by rater interaction, genotype by replication nested in year, and rater by genotype by replication nested in year were all treated as random effects. After the fixed‐effect model was used to estimate the genotype BLUEs, the data was fitted to a second set of models that included the same model terms just described but treated all terms, including genotypes, as random effects. These models were used to estimate variance components and those variance components were used to estimate trait heritability within and across years for the 7‐day and 14‐day ratings. Heritability was calculated as the “immediate response to selection” according to Holland et al. (2003).

### 2.2 GWAS and pathway analyses

Genotyping of the inbred lines in the panel was done via Genotype by Sequencing (GBS) according to Elshire et al. (2011). Briefly, SNPs were extracted from raw GBS data using the Java pipeline for GBS Bioinformatics (Glaubitz et al., 2014). SNPs were aligned against the B73 reference genome, version 4. The GBS marker dataset was imputed and filtered by removing SNPs with a minor allele frequency (MAF) <6% and any SNP with >2 alleles, resulting in a total of 1,105,817 SNPs. Useful GBS data was successfully obtained from 281 of the inbred lines, which were used in the GWAS. A subset consisting of 8000 SNPs with a low missing data rate (<7.5%) and a balanced allele frequency (MAF > 40%) was extracted for calculation of population sub‐structure (Q matrix) and linkage disequilibrium, and 148,000 unimputed SNPs were used to calculate the K matrix. K, Q, and LD were calculated in the same manner reported in Warburton et al. (2015). The software package TASSEL 3.0 (Bradbury et al., 2007) was used to perform the GWAS using the BLUE values of the 7‐day and 14‐day FAW ratings within and across years. The general linear model (GLM) was run, as well as the mixed linear model (MLM; Yu et al., 2006) using three subpopulations (Q matrix) and the K matrix. We chose a significance threshold cut‐off of *p* value ≤ 1/*N*, where *N* is total number of genome‐wide SNPs. This is less strict than the Bonferroni test of *p* value ≤ 0.05/*N*, which may be overly restrictive for GWAS (Yang et al., 2011).

The PAST (Thrash et al., 2020a) was used to perform a metabolic pathway analysis, as was first described in Tang et al. (2015). Linkage disequilibrium values between each marker and the 50 closest up‐ and downstream SNPs, and the SNP‐trait association values for significance (*p*), correlation (R^2^ or the proportion of the phenotypic variation accounted for), and allele effect as calculated by TASSEL were used by the PAST program to calculate the running enrichment score for all annotated maize pathways. A running enrichment score measures the probability that each pathway is associated with FAW resistance. The PAST program assigned each SNP to an annotated gene, based on user defined linkage disequilibrium and physical distance values of *r^2^ >* 0.8 and ±1 Kb, respectively. Each gene was assigned to a pathway using the gene annotation files in MaizeCyc (Monaco et al., 2013). Only pathways with five or more mapped genes (298 pathways) were considered in the analysis. More detail of the pathway analysis can be found in Warburton et al. (2017) and Li et al. (2019).

### 2.3 Targeted metabolite confirmation analysis

To test differential production of metabolites identified via PAST, leaves of the 31 most resistant lines in the study and 28 of the most susceptible lines based on the 7‐day visual ratings from 2019 and 2020 were collected in 2021. Least and most susceptible lines used are indicated in Table S1 in green or red highlight, respectively. Leaf tissue was collected from 10 plants of two replications of each genotype after the 14‐day damage rating scores had been collected. Leaves from each plot of each selected genotype (except Mp704 and Mp707, which had too few live plants in the field) were harvested and flash frozen in liquid nitrogen and then used in a confirmation study whereby carotenoids and chlorophylls were extracted and analyzed for preferential abundance between the selected lines. This was done as previously described (Wojdyło et al., 2021) with the modification that magnesium carbonate (MgCO_3_, 50 mg) was added to lyophilized leaf samples (500 mg) to prevent isomerization. The samples were then extracted with 5 mL hexane:acetone:methanol (2:1:1, v/v/v) containing 1% butylated hydrotoluene for 1 h in the dark on an orbital shaker (5000 rpm). The extracts were filtered through cotton plugs and the filtrates were concentrated via speedvac (Savant, Thermo Scientific). Each extract was re‐dissolved in methanol (1 mL) and particulates were removed via centrifuge.

For carotenoid and chlorophylls analysis, the re‐dissolved, centrifuged extracts were analyzed on a Waters Acquity UPLC system coupled to a Waters Xevo G2 XS Quadrupole Time‐of‐Flight (QTOF) mass spectrometer (MS). The QTOF MS was equipped with a Z‐spray ionization source running in ESI+ mode using MassLynx 4.2 software with the following settings: source temperature: 100°C, desolvation temperature: 250°C, desolvation gas flow: 600 L/h, cone gas flow: 50 L/h, capillary voltage: 3.0 kV, sampling cone voltage: 40 V. Analyses were performed in sensitivity and continuum mode, with a mass range of *m*/*z* 50–1200 and a scan time of 0.1 s. A data‐independent acquisition method with elevated collision energy (MSE) was used with 6 eV low energy and a high energy ramp from 15 to 45 eV. Separation was performed on a Waters BEH C18 1.7 μm, 2.1 × 50 mm column with the following gradient solvent system: (0.5 mL/min, solvent A: 0.1% formic acid in water; solvent B: 7:3 acetonitrile:methanol): 75% B (0.0–0.6 min), gradient to 95.1% B (0.6–6.5 min), gradient to 100% B (6.5–13.6 min), 100% B (13.6–14.6 min), then column equilibration to 5% B (14.6–17.5 min). Data were analyzed on Waters UNIFI 1.9.4 software using the “Quantify Assay Tof 2D” analysis method with lock mass (leucine‐enkephalin) corrected by UNIFI. Analytical standards (lutein and zeaxanthin) were purchased from ChromaDex and used to confirm identity of analytes and generate standard curves. Lutein and zeaxanthin are reported in μg carotenoid/g leaf sample. Relative amounts of chlorophyll a, chlorophyll b, and pheophytin b are reported as intensity counts/g leaf sample.

Normality of distribution on the metabolite levels was tested using QQ plots and by performing a Shapiro–Wilk test in R (Team, 2015). To achieve normality, metabolite content levels were transformed using log base 10. Normalized metabolite data and FAW ratings for day 7 and 14 from the 2021 field season were the input variables used to perform a correlation analysis using hierarchical clustering with 0.095 confidence intervals and 0.05 cut‐off *p* value in R. The data was scaled then used to generate a dendrogram by applying the Euclidean distance method and clustered by using the complete agglomeration method in R. Principal component analysis was performed using the same input variables used for the dendrogram. The first two PCs were used to create a PCA plot and variance by variable and individual was extracted from the analysis. Transformed and scale variables were plotted in relation to the cluster identity.

## 3 RESULTS AND DISCUSSION

### 3.1 Variation for FAW damage in the GWAS panel

There was a wide range of variation in the damage ratings on the 289 inbred lines in the study, from a low of 2.01 to a high of 7.65 over the three years (Table S1). The data over the three years of the study were well correlated, with correlations of 0.68 (2019 vs. 2021), 0.70 (2020 vs. 2021), and 0.72 (2019 vs. 2020). The 7‐day ratings were also correlated with the 14‐day ratings (0.94) (data not shown). The 31 most resistant inbred lines are highlighted in Table S1 in green and include many lines from the USDA ARS CHPRRU (19 breeding lines and released inbred lines designated Mp); CIMMYT (10 released inbreds designated CML); and NCSU (1 inbred designated NC). Considering the large effect QTL identified in some of the CPHRRU lines in previous mapping studies (Brooks et al., 2007; Womack et al., 2018, 2020), and the strong resistance demonstrated by these lines in this study (all had average damage ratings of less than 4.0 over the three years), these lines would make excellent donor lines for introgression breeding of FAW resistance into elite but susceptible maize lines. The range of phenotypic variation was also sufficient for a successful GWAS study. Entry‐mean heritability was estimated from the relevant variance components and presented in Table S3. Within years, heritability ranged from 0.68 (2019) to 0.85 (2020 and 2021) for the 7‐day ratings and 0.79 (2019) to 0.90 (2021) for the 14‐day ratings. The heritability across all three years was 0.89 for the 7‐day ratings and 0.90 for the 14‐day ratings. These results indicated a slight increase in precision in the 14‐day ratings compared to the 7‐day ratings, but the relatively high heritability across all three years for both rating scales indicated that the phenotypes were measured with sufficient precision to support the GWAS study.

### 3.2 Genes associated with FAW damage ratings

When the GLM was used for association analysis, the observed −Log10(*p* values) were higher than the expected, as can be seen in the QQ plot in Figure S1a. The observed values were much closer to the expected when the MLM was used (Figure S1b), and thus the MLM results are presented here and were used in the subsequent pathway analysis. Because of missing data of some SNPs, several of the most significant SNPs ended up comparing only one or two individuals with one allele against one or more hundreds with the other allele. To avoid this statistically risky analysis, the MAF was increased to 0.09 and the GWAS run again with 940,585 remaining SNPs. Only results of this second analysis are presented here and were used in the pathway analysis. There were 3 SNPs, corresponding to 2 linked loci, significantly associated at p < 1.06 × 10^−6^ with the data for FAW damage at 7 days after infestation, and 12 SNPs, corresponding to 5 linked loci, with the data at 14 days (Table 1). There was very good correspondence with the SNPs found to be associated in the 7‐day and 14‐day ratings. All three significantly associated SNPs in the 7‐day rating analysis were associated in the 14‐day analysis at the *p* < 10^−5^ or better, and all but one of the 12 SNPs significantly associated in the 14‐day rating analysis were associated in the 7‐day analysis at the *p* < 10^−5^ or better. The table of MLM association scores for *p* < 10^−5^ can be found in Table S4. Associations that are better than the predetermined threshold of *p* < 1.06 × 10^−6^ are highlighted in yellow.

**TABLE 1:** The 15 SNPs (listed by chromosome [Chr] and position along the chromosome) significantly (*p* < 1.06 × 10^−6^) associated with fall armyworm (FAW) damage levels measured at 7 and at 14 days after infestation were in or closely linked to 7 genes. Genes were found using G‐Browse function of MaizeGDB (Woodhouse et al., 2021). Association values (degrees of freedom, df), F values, *p* values, and Error degrees of freedom (Error df) as well as correlations (Marker R^2^), gene identifications (“Linked gene”), and annotations (“Gene name or function”) are noted

Chr	Position	df	F	*p*	Error df	Marker R^2^	Linked gene	Gene name or function
**7 Day FAW damage rating**								
1	46380400	2	16.277	2.30E‐07	252	0.041	Zm00001d028785	Not annotated
6	85713720	1	29.505	7.41E‐07	76	0.100	Zm00001d036360	cyc3 ‐ cyclin3
6	85715213	2	14.574	1.03E‐06	255	0.037		
** 14 Day FAW damage rating**								
Chr	Position	df	F	p	Error df	Marker R^2^	Linked gene	Gene name or function
4	15980038	2	18.029	6.54E‐08	201	0.058	Zm00001d049099	pdi1 ‐ protein disulfide isomerase1
4	15980032	2	18.029	6.54E‐08	201	0.058		
1	177103292	2	16.568	2.08E‐07	216	0.046	Zm00001d031097	Not annotated
8	22544824	2	16.246	2.29E‐07	261	0.039	Zm00001d008850	LEA membrane bound glycoprotein
3	139450676	1	31.336	7.46E‐07	59	0.123	Zm00001d041826	Nucleus associated protein kinase domain containing
3	139450701	1	31.336	7.46E‐07	59	0.123		
3	139450683	1	31.336	7.46E‐07	59	0.123		
3	139450697	1	31.336	7.46E‐07	59	0.123		
3	139450106	1	25.460	9.14E‐07	236	0.033		
3	139450107	1	25.460	9.14E‐07	236	0.033		
3	139450108	1	25.460	9.14E‐07	236	0.033		
5	191019633	2	14.932	8.27E‐07	227	0.040	Zm00001d017264	Cell wall‐associated receptor kinase‐like 8

The 15 SNPs associated with FAW damage levels were in or closely linked to 7 genes (Table 1), two of which were not annotated. The other five included a cyclin with kinase activity to promote cell division (cyc3, Zm00001d036360); a protein disulfide isomerase member of the thioredoxin superfamily involved in starch synthesis (pdi1, Zm00001d049099); a late embryogenesis abundant (LEA) hydroxyproline‐rich member of the glycoprotein family that is associated with the plasma membrane (Zm00001d008850); a protein kinase domain containing protein located in the nucleus (Zm00001d041826); and a cell wall‐associated receptor kinase‐like 8 (Zm00001d017264). Three of these genes (Zm00001d028785, Zm00001d041826, and Zm00001d049099) were previously found to increase expression following exposure to fungal pathogens (Hoopes et al., 2019; Swart et al., 2017). Although none of the genes were part of annotated maize pathways, one (Zm00001d049099) is a thioredoxin, and the thioredoxin pathway was found to be associated with FAW damage levels (see later).

### 3.3 Pathways associated with FAW damage levels

The pathway analysis was run on the 7‐day damage and the 14‐day damage scores separately. Pathways that were associated with increasing the trait values, and with decreasing them, were both analyzed; thus, we ran four separate pathway analyses on FAW damage. Pathways associated with reduced FAW damage scores at the (*p* value ≤ 0.05) level were identified in the 7‐day and 14‐day ratings datasets, and pathways associated with increased FAW damage scores were run on the ratings datasets collected at 7 and 14 days. Following these pathway analyses, 31 pathways were associated with increased FAW damage: 26 with the 7‐day ratings, 18 with the 14‐day ratings, and 5 in common. Another 42 pathways were associated with decreased FAW damage: 29 with the 7‐day ratings, 22 with the 14‐day ratings, and 9 in common (Table S5). There were no common pathways between the analyses for increased damage versus decreased damage, but three related pathways were found between the increased and decreased analyses. Triacylglycerol biosynthesis was associated with reduced FAW damage, while triacylglycerol degradation was associated with increased damage; phospholipid biosynthesis was associated with increased damage, and phospholipid conversion to another compound was associated with reduced damage. On the other hand, UDP‐β‐l‐arabinose biosynthesis was associated with both increased and decreased damage (via different pathways).

Some pathways share genes, and one or both pathways may become significantly associated with reductions in FAW damage scores. Which causes the reduction (or if both do) may not be readily apparent. For example, the top pathway for damage scores collected on both 7 and 14 days is the sporopollenin precursors biosynthesis pathway (32 genes total), at *p* value ≤ = 0.001 and 0.006, respectively, but the suberin biosynthesis pathway (55 genes total) is also significantly associated with a reduction in FAW damage rated at 14 days, at *p* value ≤ 0.05, and the two pathways have 26 genes in common. The cutin biosynthesis pathway (42 genes) is significantly associated with reduced damage at 7 days, and cutin and sporopollenin precursor biosynthesis pathways also have 26 genes in common. However, suberin biosynthesis is not associated with reduced damage at 7 days and cutin is not associated with reduced damage at 14 days. All three pathways could plausibly be associated, but sporopollenin precursors (which are carotenoids and carotenoid esters) may have better statistical evidence for association. Most pathways with shared genes create related compounds, such as homoserine and l‐homoserine biosynthesis. Most pathways contained several genes that jointly contributed to association with the trait. Few pathways had only one gene contributing to the trait and these, such as acyl‐CoA hydrolysis, generally had only a few genes in the pathway so statistical evidence that they are actually associated is lower.

**FIGURE 1:**
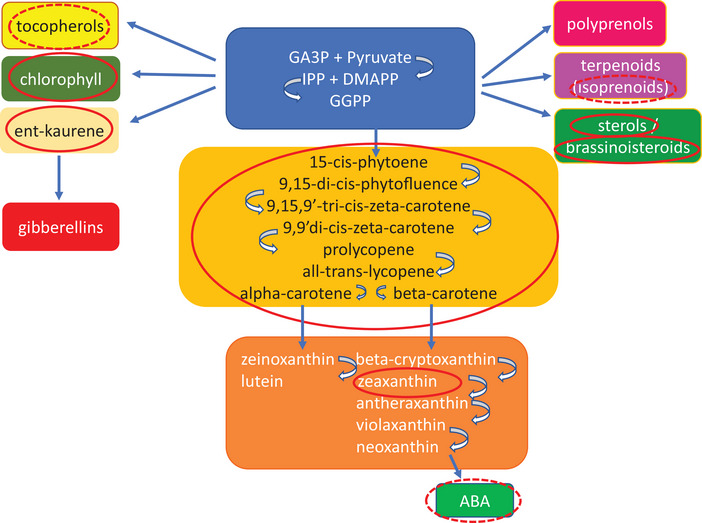
Many of the pathways associated with fall armyworm leaf damage rating scores were in the carotenoid biosynthesis pathway or pathways branching off from or downstream of it. Pathways significantly associated with damage scores are circled in red (solid line circles *p* value ≤ 0.02; dashed line *p* value ≤ 0.05).

Looking at the 31 pathways associated at a more stringent significance threshold of *p* value ≤ 0.02 (Table 2), mechanisms that may be highlighted by the current study as involved with FAW feeding resistance include the biosynthesis of carotenes including zeaxanthin and other pathways that also utilize geranylgeranyl pyrophosphate, including ent‐kaurene and chlorophyll (Figure 1). These are highly associated with FAW damage levels, both increasing and decreasing. Other mechanisms of resistance may include hormone signaling, as the IAA and sterol biosynthesis (of which brassinosteroids are a subset) as well as the ent‐kaurene biosynthesis pathway (which can be converted to gibberellins, Figure 1) were all identified as associated with damage levels. Reactions associated with FAW damage (Table 2) include the S‐adenosyl‐l‐methionine cycle, which was associated with increased damage; acyl‐CoA hydrolysis (decreasing damage); and thioredoxins and glutathione redox reaction (related reactions, associated with decreased damage). The production of lipids and waxes, including cuticular wax, phosphatidylethanolamine, and phospholipids (compounds in biological membranes) and the essential oil linalool and β‐caryophyllene), all associate with increased levels of damage. The biosynthesis of several other compounds is associated with decreased FAW feeding damage, including ascorbate, flavin or riboflavin, myo‐inositol and phytate, and the amino acid cysteine. Some compounds associated with an increase in damage ratings include ammonia, 1,4‐dihydroxy‐2‐naphthoate, the phenolic compound coumarin, and the amino acids homoserine, methionine, and homocysteine.

**TABLE 2:** The 31 pathways associated (*p* value ≤ 0.02) with fall armyworm leaf damage rating scores at 7 and 14 days after infestation, including pathways that increased and that decreased resistance in maize plants. Pathway identification (Pathway ID) from MaizeCyc, the pathway name, and association values (*p* and *q* values), the number of genes in the pathway (# genes), and notes about each pathway are included in the table

Pathway ID	Pathway name	*p* value	*q* value	# genes	Pathway notes
**Decreasing damage at 14 days after infestation**					
PWY‐6733	Sporopollenin precursors biosynthesis	0.0064	0.8666	32	Sporopollenin precursors are carotenoids and carotenoid esters
R‐ZMA‐1119348.1	Ent‐kaurene biosynthesis	0.0066	0.8666	4	Ent‐kaurene is formed from geranylgeranyl‐PP, which is also a precursor to carotenoids
R‐ZMA‐1119516.1	Trehalose biosynthesis I	0.0107	0.8666	5	Biosynthesis of a non‐typical sugar
PWY‐5148	Acyl‐CoA hydrolysis	0.0180	0.8666	6	Acyl‐CoA is a group of coenzymes that metabolize fatty acids
** Increasing damage at 14 days after infestation**					
Pathway ID	Pathway name	*p* value	*q* value	# genes	Pathway notes
CAROTENOID‐PWY	Superpathway of carotenoid biosynthesis	0.0017	0.7682	6	Carotenoids have photoprotective, antioxidant and pigmentation properties; are hormone precursors, can attract insect pollinators and indicate ripeness.
PWY‐5791	1,4‐Dihydroxy‐2‐naphthoate biosynthesis II	0.0049	0.9173	10	1,4‐Dihydroxy‐2‐naphthoic acid is converted to menaquinone (vitamin K2)
PWY‐5944	Zeaxanthin biosynthesis	0.0078	0.9173	5	Zeaxanthin is one of the most common carotenoids in nature
CHLOROPHYLL‐SYN	3,8‐Divinyl‐chlorophyllide a biosynthesis I	0.0108	0.9173	12	This component is an important intermediate in the biosynthesis of all chlorophylls
PWY‐282	Cuticular wax biosynthesis	0.0118	0.9173	12	Cuticular wax can increase resistance to leaf feeding insects
PWY‐5698	Allantoin degradation to ureidoglycolate II	0.0137	0.9173	5	Ureide metabolism in C3 and C4 plants to recycle N for growth maintenance
HOMOSERSYN‐PWY	L‐homoserine biosynthesis	0.0177	0.9173	9	Biosynthesis of amino acid
R‐ZMA‐1119374.1	Abscisic acid biosynthesis	0.0469	0.9173	5	ABA is involved in plant hormone signaling
** Decreasing damage at 7 days after infestation**					
Pathway ID	Pathway name	*p* value	*q* value	# genes	Pathway notes
PWY‐6733	Sporopollenin precursors biosynthesis	0.0012	0.3082	32	Sporopollenin precursors are carotenoids and carotenoid esters
PWY3DJ‐35471	l‐ascorbate biosynthesis (partial pathway)	0.0013	0.3082	8	Biosynthesis of vitamin C
CYSTSYN‐PWY	l‐cysteine biosynthesis I	0.0068	0.6644	10	Biosynthesis of amino acid
R‐ZMA‐1119370.1	Sterol biosynthesis	0.0097	0.6644	5	Sterols are involved in plant hormone signaling
HISTSYN‐PWY	l‐histidine biosynthesis	0.0109	0.6644	8	Biosynthesis of amino acid
R‐ZMA‐1119379.1	Flavin biosynthesis	0.0126	0.6644	6	Flavins are known antibiosis agents
R‐ZMA‐1119378.1	Myo‐inositol biosynthesis	0.0128	0.6644	4	Myo‐inositol creates some secondary messengers and is a component of structural lipids. Phytic acid is the principal storage form of phosphorus in plants.
R‐ZMA‐1119434.1	Lipid‐independent phytate biosynthesis	0.0128	0.6644	4	Related to Myo‐inositol biosynthesis
THIOREDOX‐PWY	Thioredoxin pathway	0.0147	0.6644	14	Thioredoxins sustain thiol‐based redox homeostasis in plant tissues to protect against insects.
PWY‐2301	Myo‐inositol biosynthesis	0.0150	0.6644	8	Myo‐inositol creates some secondary messengers and is a component of structural lipids. Phytic acid is the principal storage form of phosphorus in plants.
R‐ZMA‐1119509.1	Histidine biosynthesis I	0.0160	0.6644	5	Biosynthesis of amino acid
R‐ZMA‐1119437.1	Glutathione redox reactions I	0.0182	0.6933	6	Related to thioredoxin function
** Increasing damage at 7 days after infestation**					
Pathway ID	Pathway name	*p* value	*q* value	# genes	Pathway notes
PWY‐5041	S‐adenosyl‐l‐methionine cycle II	0.0033	0.7935	6	S‐adenosyl‐methionine (SAM) is a ubiquitous methyl donor and a precursor in the biosynthesis of ethylene, polyamines, biotin, and nicotianamine in plants
R‐ZMA‐1119486.1	IAA biosynthesis I	0.0075	0.7935	19	IAA is involved in plant hormone signaling
R‐ZMA‐1119501.1	S‐adenosyl‐l‐methionine cycle	0.0103	0.7935	5	S‐adenosyl‐methionine (SAM) is a ubiquitous methyl donor and a precursor in the biosynthesis of ethylene, polyamines, biotin, and nicotianamine in plants
PWY‐5669	Phosphatidylethanolamine biosynthesis I	0.0130	0.7935	8	Phospholipids in biological membranes
PWY‐6275	β‐caryophyllene biosynthesis	0.0140	0.7935	5	β‐caryophyllene biosynthesis is a natural bicyclic sesquiterpene that is a constituent of many essential oils
PWY‐5176	Coumarin biosynthesis (via 2‐coumarate)	0.0162	0.7935	8	Coumarins are known antibiosis agents and are upstream of flavonoid synthesis
PHOSLIPSYN2‐PWY	Superpathway of phospholipid biosynthesis II	0.0164	0.7935	11	Phospholipid component of biological membranes
METHIONINE‐DEG1‐PWY	l‐methionine degradation I (to l‐homocysteine)	0.0172	0.7935	4	Biosynthesis of amino acid
PWY‐6927	Chlorophyll a degradation II	0.0177	0.7935	8	Breakdown of chlorophyll in plant cells

### 3.4 Validation of metabolites in resistant versus susceptible maize lines

Using 29 of the most resistant and 28 of the most susceptible maize lines in this study, we sought correlations between the presence of zeaxanthin, ß‐carotene, lutein, chlorophyll a, chlorophyll b, and pheophytin b, with 7‐ and 14‐day damage rating. The carotenoids were measured because of the multiple carotenoid‐related pathways identified by PAST, as were the chlorophylls and pheophytins. Pheophytins are identical in structure to chlorophylls but lack magnesium(II). Chlorophylls can be degraded to pheophytins under low pH or enzymatically. Along with chlorophylls and ß‐carotene pheophytins are also involved in photosynthesis (photosystem II). We were unable to find measurable levels of zeaxanthin in the leaf samples, which is unsurprising as this compound tends to be produced in maize grains. How the biosynthesis pathway is then associated with reduced leaf feeding by FAW is thus unknown. The four other metabolites measured showed phenotypic variation among the maize lines tested, although about 80% of the lines showed low levels, with less than 300 μg/g of lutein, 2000 μg/g of ß‐carotene, 3E6 counts/g of chlorophyll a, 2E6 counts/g of chlorophyll b, and 2E6 counts/g of pheophytin b (Table S6).

**FIGURE 2:**
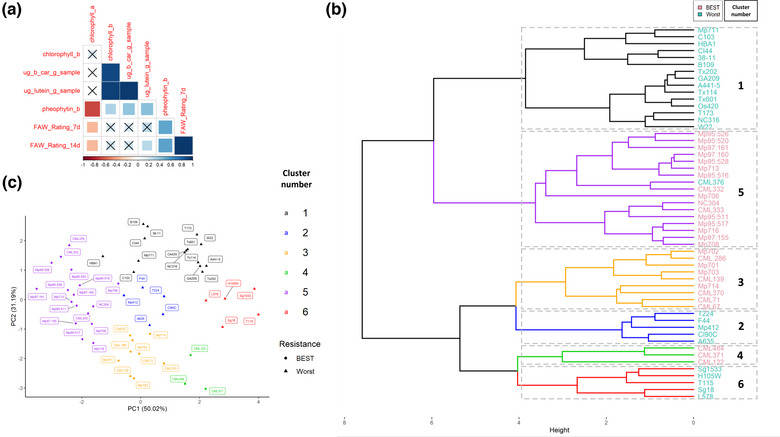
Targeted metabolite analysis for lutein, β‐carotene, chlorophyll a and b, and pheophytin b. (a) Correlation matrix, (b) dendrogram, and (c) principal component analysis (PCA) of metabolite and fall armyworm rating analysis. In the dendrogram, maize lines are colored salmon (BEST) and blue (WORST) representing resistance phenotype. In the dendrogram and PCA, clusters are represented with the following colors, 1 = black, 2 = blue, 3 = yellow, 4 = green, 5 = purple, and 6 = red. In the correlation matrix graph, blue represents positive correlation, red negative correlation, and black “X” indicates that the comparison was not significantly correlated with a *p* value cut‐off of 0.05. In the PCA, shape of points represents resistant phenotype, circle (BEST), and triangle (WORST).

Levels of these four metabolites were correlated with each other, and some with damage ratings (Figure 2a). Lutein and pheophytin b were correlated with an increase in FAW damage, and chlorophyll a was correlated with a decrease in FAW damage, in agreement with pathway PWY‐6927 (chlorophyll a degradation II) being associated with increased FAW damage ratings at 7 days (and less significantly so at 14 days, along with pathway PWY‐5098, chlorophyll a degradation I) (Table S5). Chlorophyll b is positively correlated to ß‐carotene, lutein, and pheophytin but negatively correlated with chlorophyll a. The chlorophyll cycle converts a to b forms of chlorophyll via a step through the intermediary 3,8‐divinyl‐chlorophyllide a; thus, it makes sense that a and b are negatively correlated and also that pathway CHLOROPHYLL‐SYN, which produces 3,8‐divinyl‐chlorophyllide a, is associated with increased damage in the PAST analysis. ß‐carotene was positively correlated to pheophytin and lutein, which were correlated to FAW damage, but ß‐carotene itself was not significantly correlated with FAW damage scores. These metabolites appear to agree with the metabolic pathway analysis that found carotenoids to be important in FAW resistance but are not conclusive.

The wide distribution of phenotype in relation to metabolite content made straightforward conclusions difficult. The cluster analysis (Figure 2b) and PCA (Figure 2c) of metabolites and FAW ratings showed six groups, formed based on FAW rankings and also metabolite content. For example, two of the clusters showed high levels of lutein content, one of which were FAW resistant and the other FAW susceptible. For a clearer breakdown of metabolite and resistance phenotype, Figure S2 shows the levels of each metabolite within each of the six clusters; it is apparent that some of the clusters are highly significantly different than the others for levels of that metabolite. Although the numbers of lines within each cluster reduce the statistical power of the analysis, it is likely that within the resistant lines, there is more than one mechanism leading to resistance, but that levels of lutein, pheophytin, and chlorophyll a in particular are almost certainly contributing to resistance (or susceptibility). Although the effect of each metabolite on resistance may not be enough to create resistant lines, they do help to validate the results of the PAST metabolic pathway analysis.

### 3.5 Pathways and genes in common with other larval insect pest resistance studies

A similar pathway analysis was done previously by this group on corn earworm (CEW; *Helicoverpa zea* (Boddie)) ear‐damage levels in maize (Warburton et al., 2017), and five of the same or very closely related pathways were identified in both studies. These include wax esters biosynthesis II, simple coumarins biosynthesis, geranylgeranyl diphosphate (GGPP) biosynthesis, the chlorophyll degradation pathway, ent‐kaurene biosynthesis I, and phospholipases biosynthesis. Wax esters are a component of epicuticular wax, which is a physical barrier to insect predation. Coumarins belong to the phenolics class of compounds, which repel feeding insects. The production of GGPP is the first step leading into the carotenoids and related pathways (Figure 1). Phospholipases may be expressed when plants are wounded, as during insect feeding, and initiate production of important defense signaling molecules, such as oxylipins and jasmonates (De Vleesschauwer et al., 2014). All these mechanisms were important in FAW resistance as well and may form part of the common defense mechanisms of maize plants against many larval feeding insects.

Many of the germplasm lines that exhibited the highest levels of resistance to FAW leaf feeding damage in the current study were developed and released by the USDA‐ARS at Mississippi State. During development, these lines were selected for resistance to both FAW and southwestern corn borer (SWCB; *Diatraea grandiosella* Dyar) leaf feeding. Germplasm lines released as sources of resistance to both insects include Mp703–708, Mp713, Mp714, and Mp716 (Williams & Davis, 1980, 1982 , 2000, 1984, 2002; Williams et al., 1990). These lines, or crosses among them, have exhibited resistance to other Lepidoptera including European corn borer (ECB; *Ostrinia nubilalis* Hubner) in field tests in Tennessee and Missouri, and sugarcane borer (SCB; *Diatraea saccharalis* (F.)) in field studies in Louisiana (Davis et al., 1998). Germplasm lines Mp701, Mp702, and Mp704 exhibited resistance to a stem borer in Africa (*Chilo partellus* (Swinhoe)) (Ampofo et al., 1986) and to Asian corn borer (*Ostrinia furnacalis* (Guenee)) in the Philippines (Bato et al., 1983). In laboratory bioassays, CEW, FAW, SWCB, SCB, and southern corn stalk borer (*Diatraea crambidoides* (Grote)) larvae reared on diets containing tissues of resistant inbred lines weighed significantly less than those reared on diets containing tissue of susceptible inbred lines (Davis et al., 1999; Williams & Davis, 1985b; Williams et al., 1985, 1987b, 1990b). Antibiosis and larval non‐preference appeared to be mechanisms of resistance to lepidopteran larvae (Williams et al., 1987a).

An array of additional studies confirmed that breeding for leaf feeding resistance to one pest improved resistance to other pests (Williams & Davis, 1982). Williams et al. (2000, 1998) suggested that the juvenile/adult vegetative phase change, which is controlled by the *glossy15* gene, is associated resistance to first generation FAW and SWCB. It was further found that a unique 33‐kD cysteine proteinase was expressed in response to feeding by FAW and larval growth was inhibited when fed with maize callus expressing proteinase (Pechan et al., 2000). Another recent study of the defense response of resistant maize lines to ECB and western corn rootworm (*Diabrotica virgifera virgifera*) used transcriptomics to identify changes in gene expression following feeding damage (Pingault et al., 2021). They found significant gene expression changes in a number of genes, some in common with the current study. These include genes encoding, regulating, or modifying carotenoids, coumarins, S‐adenosyl‐l‐methionine, methionine, acyl‐CoA, glutathione, thioredoxin, histidine, myo‐inositol, flavin, sterol, trehalose, chlorophyll, phospholipases, and phospholipids. While this list is too long and varied to point to a specific common mechanism of resistance, it does suggest that common mechanisms may exist, and may indicate where the search may begin. Results of both field and laboratory experiments indicate that the lines developed and released by the USDA‐ARS exhibit resistance to multiple insects. Genes identified in the current study, via GWAS and pathway analysis, can be introgressed via marker assisted selection (MAS) into susceptible maize lines to verify their utility to boost native host plant resistance in field conditions, and develop new resistant hybrids.

## CONFLICT OF INTEREST

The authors declare no conflict of interest.

## AUTHOR CONTRIBUTIONS

**Marilyn L. Warburton**: Conceptualization; formal analysis; investigation; methodology; project administration; resources; software; writing–original draft. **Sandra W. Woolfolk**: Investigation; methodology; writing–review and editing. **J. Spencer Smith**: Data curation; formal analysis; methodology; writing–review and editing. **Leigh K. Hawkins**: Conceptualization; data curation; methodology; writing–review and editing. **Lina Castano‐Duque**: Validation; visualization; writing–original draft; writing–review and editing. **Matthew D. Lebar**: Validation; visualization; writing–original draft; writing–review and editing. **W. Paul Williams**: Funding acquisition; project administration; resources; supervision; writing–review and editing.

## Supporting information


**Supplemental Figure 1**: QQ plot of association values calculated with the General Linear Model (GLM; 1a) and Mixed Linear Model (MLM; 1b).

**Supplemental Figure 2**: Box plots showing clusters and the distribution of the amounts of metabolites and Fall Armyworm ratings. Box‐plot whiskers depict the maximum and minimum without outliers, and the box depicts median, first and third quantiles distribution. Data shown is transformed and scale to center around 0.

**Supplemental Table 1**: Fall Armyworm damage scores at 7 and 14 days after infestation for the panel of 289 diverse maize inbred lines used for this study, including averages and standard deviations over replications within years, and averaged over years. The lines used in the metabolite validation study are highlighted in green (most resistant) and red (least resistant).

**Supplemental Table 2**: Fall Armyworm damage score descriptions, from the original rating scale of Davis et al., 1991, for 7‐day and 14‐day time points following infestation with Fall Armyworm (FAW) neonates.

**Supplemental Table 3**: Variance components, standard errors (SE), and heritability (H^2^) within and across years, for 7‐day and 14‐day ratings.

**Supplemental Table 4**: The MLM association scores for SNPs associated with Fall Armyworm (FAW) damage levels (*p* value ≤ 0.05).

**Supplemental Table 5**: The pathways associated with Fall Armyworm (FAW) damage levels (*p* value ≤ 0.05).

**Supplemental Table 6**: Averaged data for the metabolites analyzed, clusters summary statistics and PCA variance contribution information.
