# Supplementary material for: Genes and genetic mechanisms contributing to fall armyworm resistance in maize
Source: Plant Genome. 2023 Mar 2;16(2):e20311. doi: 10.1002/tpg2.20311 (PMC12806859; doi:10.1002/tpg2.20311)

Supplemental Figure 1a: QQ plot of the General Linear Model used for association analysis, with the observed -Log10(p values) (y axis) compared to the expected -Log10(p values) (x axis).

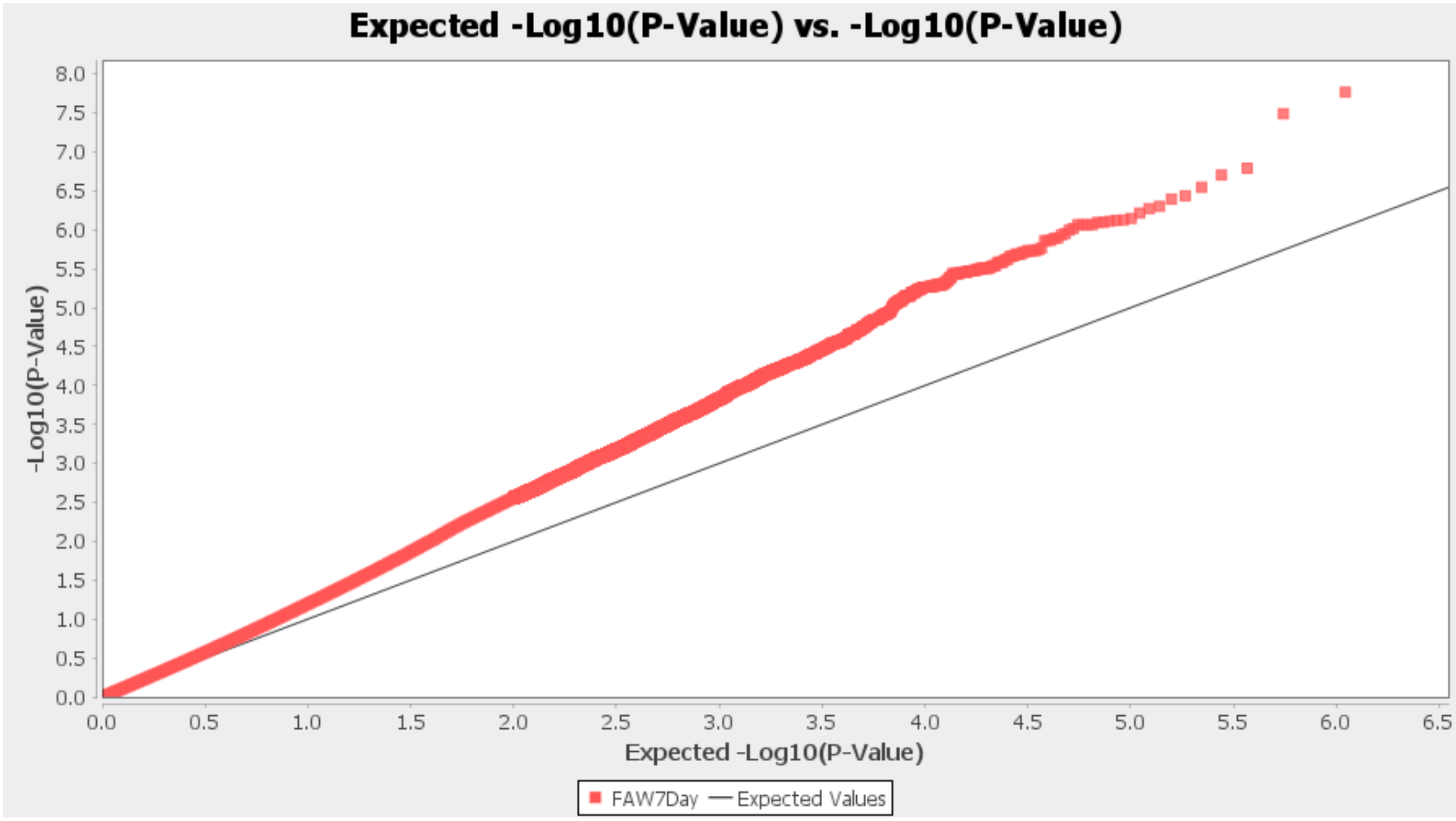


Supplemental Figure 1b: QQ plot of the Mixed Linear Model used for association analysis, with the observed -Log10(p values) (y axis) compared to the expected -Log10(p values) (x axis).

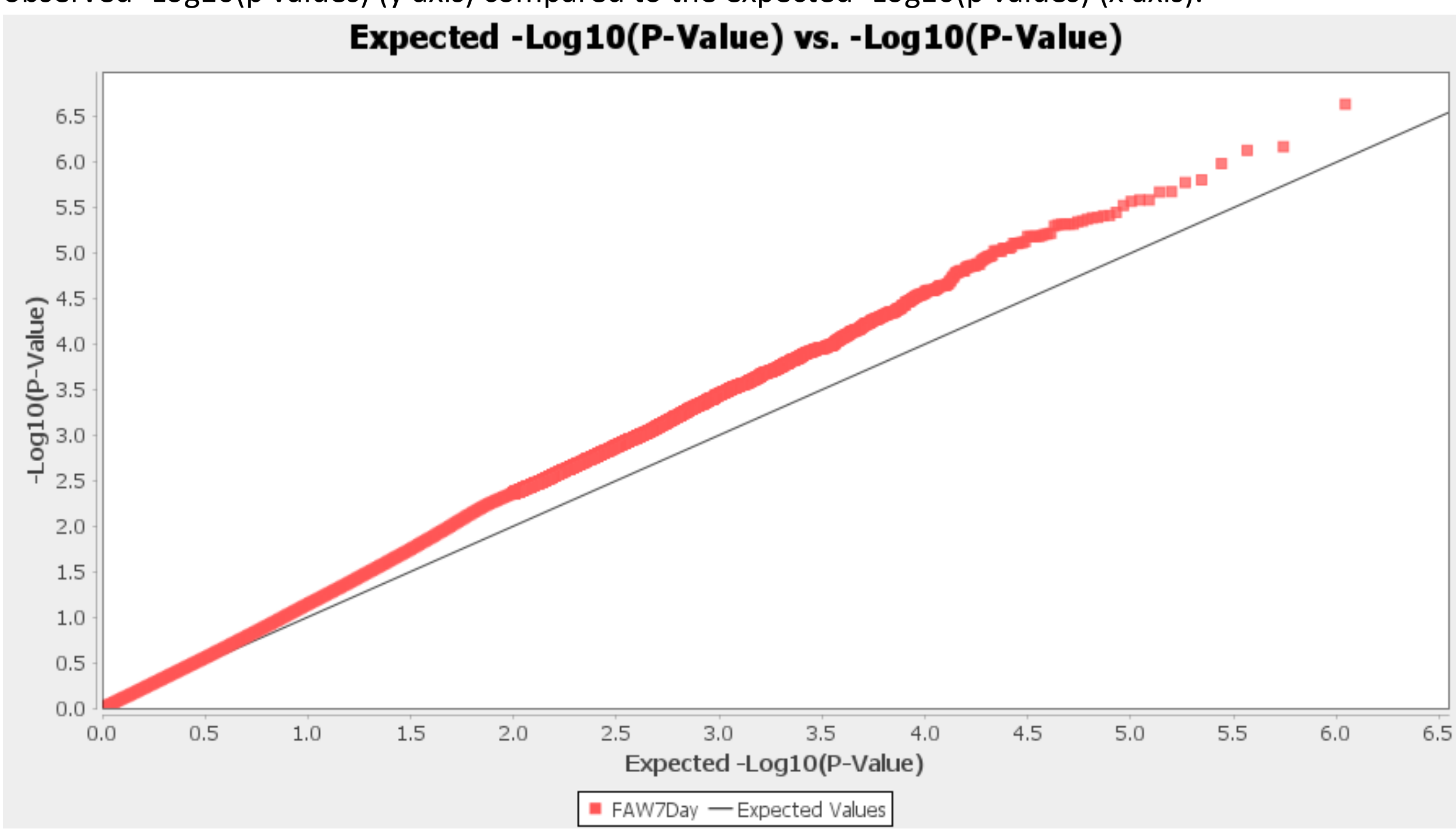

Supplement: Supplementary file 1 [file TPG2-16-e20311-s001.pdf]
