# Supplementary material for: Genes and genetic mechanisms contributing to fall armyworm resistance in maize
Source: Plant Genome. 2023 Mar 2;16(2):e20311. doi: 10.1002/tpg2.20311 (PMC12806859; doi:10.1002/tpg2.20311)
Supplement: Supplementary file 2 [file TPG2-16-e20311-s006.pdf]

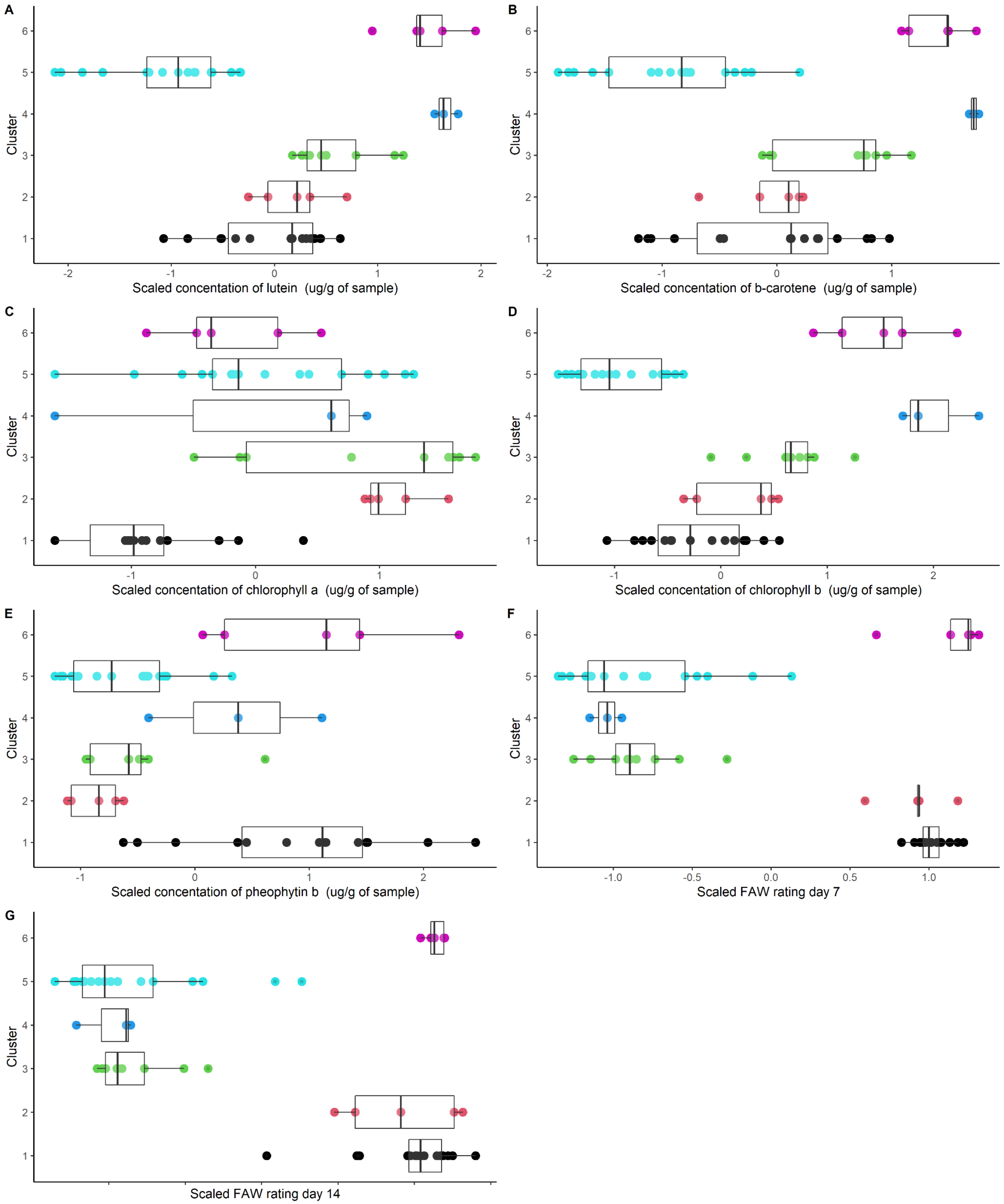
A
B
C
D
E
F
G
Cluster
Scaled concentation of lutein (ug/g of sample)
Scaled concentation of b-carotene (ug/g of sample)
Scaled concentation of chlorophyll a (ug/g of sample)
Scaled concentation of chlorophyll b (ug/g of sample)
Scaled concentation of pheophytin b (ug/g of sample)
Scaled FAW rating day 7
Scaled FAW rating day 14
