# Supplementary material for: Genes and genetic mechanisms contributing to fall armyworm resistance in maize
Source: Plant Genome. 2023 Mar 2;16(2):e20311. doi: 10.1002/tpg2.20311 (PMC12806859; doi:10.1002/tpg2.20311)
Supplement: Supplementary file 3 [file TPG2-16-e20311-s004.docx]

**Supplemental Table 3a**: Variance components, standard errors (SE), and heritability (H^2^) within and across years, for 7-day ratings when genotypic effects are modeled as random effects.

|  | **2019** | | **2020** | | **2021** | |  |  | **3-Year** | |
| --- | --- | --- | --- | --- | --- | --- | --- | --- | --- | --- |
| **Variance Component** | **Estimate** | **SE** | **Estimate** | **SE** | **Estimate** | **SE** |  | **Variance Component** | **Estimate** | **SE** |
| Rep | 0.520 | 0.739 | 0.020 | 0.030 | 0.117 | 0.169 |  | Year | 0.000 | . |
| Genotype | 0.822 | 0.106 | 1.090 | 0.108 | 1.146 | 0.125 |  | Rep(Year) | 0.136 | 0.087 |
| Genotype*Rep | 0.719 | 0.065 | 0.318 | 0.031 | 0.572 | 0.052 |  | Genotype | 0.937 | 0.089 |
| Residual | 0.768 | 0.013 | 0.568 | 0.009 | 0.485 | 0.008 |  | Genotype*Year | 0.091 | 0.027 |
| H^2^ | 0.68 |  | 0.85 |  | 0.85 |  |  | Genotype*Rep(Year) | 0.536 | 0.029 |
|  |  |  |  |  |  |  |  | Residual | 0.604 | 0.006 |
|  |  |  |  |  |  |  |  | H^2^ | 0.89 |  |

**Supplemental Table 3b:** Variance components, standard errors (SE), and heritability (H^2^) within and across years, for 14-day ratings when genotypic effects are modeled as random effects.

|  | **2019** | | **2020** | | **2021** | |  |  | **3-Year** | |
| --- | --- | --- | --- | --- | --- | --- | --- | --- | --- | --- |
| **Variance Component** | **Estimate** | **SE** | **Estimate** | **SE** | **Estimate** | **SE** |  | **Variance Component** | **Estimate** | **SE** |
| Rep | 0.028 | 0.146 | 0.060 | 0.090 | 0.183 | 0.191 |  | Year | 0.044 | 0.108 |
| Rater | 0.074 | 0.203 | 0.026 | 0.040 | 0.000 | . |  | Rep(Year) | 0.107 | 0.090 |
| Rep*Rater | 0.114 | 0.161 | 0.004 | 0.006 | 0.013 | 0.011 |  | Rater | 0.016 | 0.039 |
| Genotype | 0.658 | 0.071 | 1.012 | 0.100 | 1.044 | 0.109 |  | Rep*Rater(Year) | 0.040 | 0.030 |
| Genotype*Rep | 0.286 | 0.030 | 0.268 | 0.029 | 0.659 | 0.044 |  | Genotype | 0.860 | 0.079 |
| Genotype*Rep*Rater | 0.070 | 0.008 | 0.087 | 0.008 | 0.071 | 0.006 |  | Genotype*Year | 0.040 | 0.018 |
| Residual | 0.795 | 0.009 | 0.716 | 0.008 | 0.846 | 0.008 |  | Rater*Year | 0.014 | 0.034 |
| H^2^ | 0.79 |  | 0.85 |  | 0.90 |  |  | Genotype*Rep(Year) | 0.460 | 0.022 |
|  |  |  |  |  |  |  |  | Geno*Rep*Rater(year) | 0.075 | 0.004 |
|  |  |  |  |  |  |  |  | Residual | 0.797 | 0.005 |
|  |  |  |  |  |  |  |  | H^2^ | 0.90 |  |
